# The Conservatism Paradox: Conservatism Among Teachers Predicts Resistance Even to Conservative Change

**DOI:** 10.5334/irsp.1041

**Published:** 2026-11-03

**Authors:** Eszter Hadnagy, Patricia Ciordas, Anna Kende

**Affiliations:** 1 Doctoral School of Psychology, ELTE Eötvös Loránd University, Budapest, Hungary; 2 Institute of Psychology, ELTE Eötvös Loránd University, Budapest, Hungary

**Keywords:** resistance to change, educational change, cognitive conservatism, ideological conservatism, identity threat, teacher

## Abstract

Hungary’s educational system has recently undergone significant conservative changes, which have met notable resistance from educators. Cognitive and ideological conservatism, as well as perceived individual and collective professional identity threat, have been linked to such resistance. However, there are gaps in understanding how conservatism and threat perception influence resistance specifically to conservative educational changes. We investigated whether teachers’ levels of conservatism and the vulnerability of their collective and individual professional identity would predict higher resistance to change. A convenience sample of 502 public school teachers from diverse educational levels and regions in Hungary completed an online survey. Using Structural Equation Modelling, we analysed the relationships among conservatism, identity threat, and resistance to change. Higher need for closure predicted greater resistance, whereas ideological conservatism showed mixed effects: right-wing authoritarianism predicted greater resistance, while institutional system justification predicted lower resistance, likely due to its palliative role in affirming the status quo. Findings suggest that teachers’ resistance to educational change is shaped by both a need for security within the policy framework and concerns over professional identity.

## Introduction

Conservative ideology is typically associated with resistance to change, a preference for stability, and support for tradition and authority. Yet paradoxes arise when resistance is directed toward ideologically conservative reforms. As Jost et al. (2003; 2009) note, such cases include right-wing revolutionaries seeking radical change to restore hierarchies, selective endorsement of authority (e.g., supporting the military but opposing redistributive policies), libertarians promoting personal freedom while endorsing market policies that increase inequality, and populists combining economic interventionism with traditional norms. Conservatives have also historically come to support reforms – such as women’s suffrage, child-labour laws, and civil-rights legislation – they once opposed, and may resist reforms they view as abrupt, illegitimate, or threatening to identity (Jost, 2017). Thus, the conservatism–change relationship is shaped not linearly but by competing epistemic, existential, and relational motives (Hennes et al., 2012). This study focuses on one such complexity: how teachers high in conservatism respond to state-led conservative educational reforms.

Starting in 2012, Hungary’s education system underwent a series of policy changes aligned with the government’s conservative ideology.^1^ Central to these reforms was the centralization of governance: responsibilities once held by local municipalities, such as school maintenance and operational decisions, were transferred to a state agency, sharply reducing schools’ and teachers’ autonomy. Teachers must complete 120 hours of state-mandated in-service training every seven years, with no choice over qualifying courses. Supervisors assign off-hours, often free or online sessions; unapproved or fee-based programs require individual payment and approval. Many view these as irrelevant burdens, making scepticism toward ‘continuous training’ a clear form of resistance to the top-down agenda. The curriculum was revised to emphasize national history, heritage, and moral education, while limiting teacher input. State-approved, standardized textbooks restricted alternative pedagogical materials, narrowing classroom critical engagement. A new evaluation system tied career advancement and pay to state-defined performance indicators. The top-down approach, with little consultation, fuelled dissatisfaction, exclusion from decision-making, and professional identity threat. Resistance included protests against centralized control, curriculum and textbook decisions made without teachers, increased administrative burdens, and the application of new salary and career systems (Györgyi, 2015).

### Resistance to educational change

Change is inevitable for all organizations, including educational institutions, to remain competitive, technologically advanced, and aligned with government policies, with globalization further increasing this necessity. Yet change often meets resistance (Kalman & Bozbayindir, 2017; Van Dam, Oreg, & Schyns, 2008), as people tend to favour tradition over progress (Jost, 2015). This is reinforced by cognitive consistency – seeking information that confirms existing views and avoiding contradictory evidence (Festinger, 1957) – and by social factors, as altering attitudes or behaviours can feel like leaving the comfort of a shared social reality (Hardin & Higgins, 1996; Jost, 2015).

### Cognitive styles and ideologies as sources of resistance to change

Cognitive style (i.e., people’s preferred way of processing information (Ausburn & Ausburn, 1978), influences attitudes toward change, including organizational change, as some styles exhibit greater resistance than others. A high need for closure reflects a desire for quick, definite conclusions to avoid uncertainty, making individuals more cognitively conservative (Kruglanski et al., 2006). Those high in need for closure tend to cling to existing beliefs and resist integrating new or conflicting information (Kruglanski et al., 1993). In a study, acceptance of a plurality of ideas – defined as the ability to shift perspectives and consider opposing viewpoints – was linked to openness to change, while a dogmatic style predicted resistance (Đurišić-Bojanović, 2016). Routine-seeking, another dispositional component, also predicted teachers’ resistance to educational change (Kalman & Bozbayindir, 2017). In contrast, need for cognition is associated with lower resistance to change (Cacioppo et al., 1996). Individuals high in need for cognition seek out and critically evaluate diverse information sources, including unfamiliar ones (Cacioppo et al., 1996; Petty et al., 2009), making them less cognitively conservative and less reliant on simple cues. These tendencies can influence resistance behaviours (Haugtvedt & Petty, 1992). Examining need for closure and need for cognition separately clarifies their unique contributions.

Not independent from cognitive styles, political and social beliefs also shape resistance to change (Jost et al., 2009; White et al., 2020). Political and ideological conservatism combine a commitment to traditional values, norms, and practices (Day & Lee, 2011) with needs for order, structure, and cognitive closure (Jost et al., 2008). Consequently, ideological conservatism correlates positively with acceptance of inequality, political conservatism, right-wing orientation, and resistance to change (Critcher et al., 2009; Golec de Zavala, & Van Bergh, 2007; Kemmelmeier, 1997) while more liberal individuals score higher in need for cognition (Hennes et al., 2012; Jost & Krochik, 2014; Sargent, 2004). Among Italian psychology students, those high in need for closure were more likely to vote for right-wing parties, hold conservative attitudes, display nationalism, and favour autocratic leadership and centralized power (Chirumbolo et al., 2004). As ideological conservatism involves defending traditional values and justifying existing systems (Jost et al., 2008), political conservatism serves as a form of system justification – motivating resistance to change and rationalizing inequality (Jost et al., 2004).

The tendency that system justification enhances ingroup favouritism among high-status groups and outgroup favouritism among low-status groups (Jost & Thompson, 2000) is also evident in education. Conservative individuals are particularly inclined toward system justification, which extends to school institutions. In the US, white, middle-class teachers and parents/caregivers – members of dominant groups whose cultural capital, values, and assumptions are embedded in the education system – often resist reforms that might alter the status quo from which they benefit (McKenzie & Scheurich, 2008; Theoharis, 2007). However, system justification theory posits that there is a motivation to uphold the system that transcends self-interest (Jost et al., 2004). Disadvantaged groups, such as low-income individuals and African Americans, may also support existing inequalities to reduce ideological dissonance (Jost et al., 2007). Strong system-justifying attitudes lead people to perceive educational policies as fair, regardless of personal benefit (Igliozzi et al., 2024), and to resist equality-promoting measures like affirmative action by legitimizing the status quo (Phelan & Rudman, 2011). In summary, system justification in education mirrors its broader societal role, fostering resistance to reform across status groups, including those disadvantaged by the system.

As right-wing authoritarianism (RWA; Altemeyer, 1981) does not simply reflect ideological conservatism but also captures the strong adherence to conventionalism and traditional norms and values, it can also manifest itself in resistance to change. In fact, as Jost et al. (2003) explain, the RWA scale primarily measures resistance to change. Furthermore, RWA is associated with the desire to achieve and maintain cognitive closure (Altemeyer, 1981; Cornelis & Van Hiel, 2006). Therefore, we can assume that RWA would be closely associated with resistance to educational reforms or institutional change. Nevertheless, the distinction between system justification and RWA is important because they represent different facets of ideological conservatism. System justification focuses on defending and legitimizing the current (Jost et al., 2004), while RWA (Altemeyer, 1981) emphasizes a hierarchical and authoritarian worldview. Measuring both provides a comprehensive understanding of how ideological conservatism influences resistance to educational change from different angles.

### Identity threat as an obstacle to accepting change

Teachers often perceive their professional roles as educators integral to their identity (Day & Lee, 2011). Government-led educational reforms often challenge and destabilize current teaching practices, increase teachers’ workloads, and frequently overlook the importance of this professional identity (Day, 2002). When such changes are experienced as threats to professional identity, either on an individual level (e.g., loss of control, devaluation of expertise) or a collective level (e.g., undermining the status of the profession), they often provoke strong emotional responses, such as fear, anxiety, and insecurity (Day & Lee, 2011). In a broader socio-political context, such responses are often rooted in a perception of the world as unstable, threatening, or dangerous – a worldview that has been linked to increased resistance to change (Duckitt, 2001). Ultimately, perceived identity threats can serve as a catalyst for resisting educational reforms, especially when they challenge core values or disrupt teachers’ sense of purpose and belonging (for the connection between identity threat and resistance to change; see Jost et al., 2007).

These epistemological foundations – particularly feelings of threat and fear – are closely tied to conservatism, linking identity threats with conservative cognitive styles and ideologies. Educational change often brings structural instability, vague expectations, or loss of control, disrupting the need for stability (Kay & Friesen, 2011). Teachers high in need for closure and low in need for cognition may view reforms as threats to professional identity, rooted in perceived loss of epistemic clarity and role stability. Prior qualitative and mixed-method studies showed that teachers who valued structure, stability, and certainty often resisted reforms that disrupted routines, roles, and authority (van Veen et al., 2005). They may also generalize this discomfort into collective threat when shared norms and procedures are undermined (Day et al., 2006).

In contrast, ideological conservatism, measured through right-wing authoritarianism (RWA) and system justification, reflects relational and system-level motives. RWA involves a strong preference for social cohesion, deference to authority, and maintenance of traditional hierarchies (Altemeyer, 1996; Duckitt & Sibley, 2007). Changes challenging these structures may be perceived as collective identity threats, particularly when teachers are excluded from decision-making, see their expertise devalued, or feel their status and autonomy eroded (Sachs, 2001). Such disruptions can also be experienced individually, as challenges to professional self-understanding when reforms contradict deeply held beliefs about teaching (Kelchtermans, 2011).

System justification similarly motivates defence of the status quo (Jost & Banaji, 1994), making institutional restructuring feel threatening to both teachers’ self-concept and the perceived stability of education. Given conservatives’ heightened sensitivity to identity threats (Jost et al., 2007), teachers high in need for closure or holding conservative views may see reforms as threats, amplifying resistance. Yet resistance is not exclusive to conservatives: when reforms reflect conservative ideals (e.g., stricter curricula or centralized governance), liberal-minded teachers may also feel threatened and oppose them (Achinstein & Ogawa, 2006). Thus, resistance can occur across the ideological spectrum, depending on whether reforms align or conflict with core beliefs. This study examines how cognitive and ideological conservatism predict teachers’ attitudes toward conservative educational changes.

### Aims and objectives

This study aimed to examine the factors influencing teachers’ resistance to conservative educational changes, focusing on perceived identity threat, cognitive conservatism (need for closure, need for cognition), and ideological conservatism (right-wing authoritarianism, institutional system justification). We addressed the paradox that while conservatism and threat perception typically predict resistance to change, the conservative direction of the reforms could instead foster support. We conducted our study testing the following hypotheses:

H1: Need for closure and right-wing authoritarianism are expected to predict higher levels of teachers’ resistance to educational change (resistance to legislative changes and resistance to continuous training), whereas need for cognition and institutional system justification are expected to predict lower levels.

H2: Higher perceived individual and collective professional identity threat will predict higher levels of teachers’ resistance to educational change.

H3: Perceived identity threat is expected to mediate the associations between conservatism and resistance.

Because professional role identification may shape threat perceptions and attitudes, we controlled for identification with the teacher role. We also controlled for political orientation, school level, and work region to ensure that hypothesised effects were not explained by these background variables.

## Methods

### Participants and procedure

The online survey study was conducted in Hungary among teachers working in the public sector. A sample size of 395 participants was determined using G*Power, ensuring sufficient statistical power to detect effect sizes above *f*^2^ = .02, with a significance level of 0.05 and a power of 0.80 (Cohen, 1988). A total of 502 participants were included in the study. Informed consent was obtained from all participants prior to their involvement in the study. The current study was part of an omnibus study using overlapping variables to test a different research question. The study was preregistered at https://aspredicted.org/SNN_5KX.

Invitations were sent via email to schools, and posts were made and distributed on social media platforms to reach potential participants. Participants filled out the survey on Qualtrics, the language of the questionnaire was Hungarian. For the survey, we utilized pre-existing translations of questionnaires and items, and in cases where they were not available, we employed a rigorous process involving independent translators and back-translators for translation. A total of 776 participants filled out the questionnaire; only those who responded to all study variables were included in the analyses. Those who did not identify as teachers, or did not work in the public sector, were excluded from the analysis. To address issues of missing data, we employed the Full Information Maximum Likelihood (FIML) method. We conducted all statistical analysis using IBM SPSS version 27.0 and MPlus software, version 8 (Muthen, Muthén, & Asparouhov, 2016).

Participants’ ages ranged from 23 to 69 years (*M* = 50.30, *SD* = 9.80). Eighteen percent of participants identified as male, 71% as female, and 1.8% chose not to specify their gender. Political orientation was measured on a 1 (left-wing) to 10 (right-wing) scale, with participants’ mean score being 5.18 (SD = 2.27), indicating a balanced distribution along the ideological spectrum.

Regarding the participants’ professions: 15.1% were primary school teachers, 25.5% secondary school teachers, 31.1% high school teachers, 12.2% special education and developmental educators, and 6.8% indicated ‘Other’. They all had university degrees.

In terms of job roles within the educational sector: 16.3% worked in elementary schools teaching in grade 1–4, 27.9% in elementary school teaching in grade 5–8, 3% in vocational schools, technical high schools, 29.5% in general high schools, 12.9% selected ‘Other’, and 1% indicated ‘I am not currently working as an educator, although I am qualified as one’.

Participants in this study were geographically diverse, with 39.6% residing in the capital city, 12.7% in county seats, 22.9% in cities, and 15.3% in municipalities.

### Measures

*Resistance to educational change* was measured using two subscales from Lomba-Portela, Domínguez-Lloria, and Pino-Juste (2022). Ten items assessed resistance to legislative changes (5 items, e.g., “With the educational changes I think ‘the cure will be worse than the disease.”) and resistance to continuous training (5 items, e.g., “The training courses do not provide me with relevant content.”). Participants rated their agreement on a 5-point Likert scale from 1 (strongly disagree) to 5 (strongly agree).

Cognitive conservatism was assessed using two scales, *Need for Closure Scale:* The Hungarian translation by Csanádi, Harsányi, and Szabó (2009) of Webster and Kruglanski’s (1994) scale, consisting of 15 items (e.g., “I don’t like situations that are uncertain.”) rated on a 6-point scale from 1 (strongly disagree) to 6 (strongly agree). Higher average scores indicate a stronger tendency to seek quick and definite answers and avoid ambiguity, whereas lower scores reflect greater tolerance for uncertainty and openness to new information. *Need for Cognition Scale:* By Cacioppo and Petty (1982), with five items (e.g., “I really enjoy a task that involves coming up with new solutions to problems.”) rated on a 4-point scale from 1 (very strong disagreement) to 4 (very strong agreement). Higher average scores indicate greater enjoyment of effortful cognitive activity, whereas lower scores suggest lower motivation to engage in such tasks.

Ideological conservatism was measured using *Right-Wing Authoritarianism Scale:* Adapted from Heller et al. (2020), with three items (e.g., “Established conducts should not be questioned.”) rated on a 5-point Likert scale.

*Institutional System Justification Scale* was developed for this study based on Kay and Jost (2003), with seven items assessing views on the Hungarian education system (e.g., “The Hungarian education system is the best in the world.”) rated on a 5-point Likert scale.

Perceived identity threat was assessed using two scales adapted from Bosson et al. (2021) and Wohl et al. (2010): *Individual Threat Scale:* five items measuring perceived threats to identity as teachers (e.g., “It is quite easy for a teacher to lose the respect that a teacher deserves.”) rated on a 5-point Likert scale. *Collective Threat Scale:* five items assessing collective feelings of threat and fear about teachers as a group (e.g., “I am worried about the future of teachers.”) rated on a 5-point Likert scale.

*Identification with the teacher role* was measured using 4 items adapted from Doosje et al. (1995) on a 5-point Likert scale (e.g., “I see myself as a teacher.”). The full wording of all adapted or newly generated scale items is provided in Supplementary Material S1.

In addition to the primary constructs, we also collected a range of background variables for example, to use as *covariates*. Teachers’ political orientation was assessed with a single item (“People often describe their political orientation as left or right; please place yourself on the following 1–10 scale from Left to Right”), teaching position (kindergarten, lower-grade elementary, upper-grade elementary, secondary/high school, university, special-needs, other), pedagogical rank (intern, Teacher I, Teacher II, master teacher, research teacher, other), current teaching level (grades 1–4, grades 5–8, vocational school, secondary/high school, other, not currently teaching), and work location (capital, county seat, city, village).

## Results

### Descriptive Statistics

In Table 1, we present descriptive statistics, scale reliability, and correlations among the study variables. Because of the rather low values of Cronbach’s alpha for both types of resistances to educational change, we conducted a confirmatory factor analysis of the original factor structure and identified acceptable model fit for the two types of resistance measures for educational changes (*χ^2^* = 94.939 (*df* = 33), *p* < .001; TLI = .92; CFI = .89; RMSEA = .06; SRMR = .05). As descriptive statistics show, participants displayed moderate levels of resistance to both types of educational changes, their responses showed a relatively high level of need for closure, while their need for cognition level was moderate. On average, participants in the study also exhibited moderate levels of right-wing authoritarianism and low levels of system justification. Participants had a moderately high level of individual identity threat, and they exhibited high levels of collective identity threat. Participants’ identification with the teacher role suggested a strong sense of identity related to their role as educators.

**Table 1:** Descriptive statistics, scale reliability and correlations between study variables.


VARIABLES	RESPONSE SCALE	MEAN (SD)	*a*	1	2	3	4	5	6	7	8

1. Resistance to legislative changes	1–5	3.83 (0.70)	.68	–							

2. Resistance to continuous training	1–5	3.58 (0.71)	.67	.33**	–						

3. Need for closure	1–6	3.93 (0.75)	.84	.12**	.26**	–					

4. Need for cognition	1–4	3.20 (0.75)	.69	.06	–.09*	–.26**	–				

5. Right-wing authoritarianism	1–5	2.42 (0.89)	.63	–.13**	.20**	.39**	–.29**	–			

6. Institutional system justification	1–5	1.67 (0.64)	.84	–.48**	–.15**	.12**	–.18**	.41**	–		

7. Individual identity threat	1–5	3.93 (0.75)	.77	.23**	.30**	.14**	.08	.04	.15**	–	

8. Collective identity threat	1–5	4.02 (0.93)	.79	.59**	.25**	.10*	.07	–.15**	.49**	.33**	–

9. Identification with the teacher role	1–5	4.12 (0.68)	.63	.14**	–0.60	.08	.13*	.01	–.07	.05	.18**


*Notes*: *p < .05; **p < .001.

Zero-order correlations showed that most study variables were correlated with resistance to legislative changes and continuous training. High need for closure was linked to greater authoritarianism, *r* = .39, *p* < .001, 95% *CI* [.31, .46]; higher institutional system justification, *r* = .12, *p* = .007, 95% *CI* [.03, .21]; greater individual identity threat, *r* = .14, *p* = .001, 95% *CI* [.06, .23]; and – more weakly – collective identity threat, *r* = .10, *p* = .030, 95% *CI* [.01, .18]. Conversely, high need for cognition correlated with lower right-wing authoritarianism, *r* = –.29, *p* < .001, 95% *CI* [–.37, –.21]; lower institutional system justification, *r* = –.18, *p* < .001, 95% *CI* [–.26, –.09]; and higher teacher-role identification, *r* = .13, *p* = .005, 95% *CI* [.04, .21]. Right-wing authoritarianism was negatively correlated with collective identity threat, *r* = –.15, *p* < .001, 95% *CI* [–.23, –.06]. Institutional system justification was negatively correlated with both individual identity threat, *r* = –.15, *p* < .001, 95% *CI* [–.23, –.06], and collective identity threat, *r* = –.49, *p* < .001, 95% *CI* [–.56, –.42], suggesting a strong link between feeling threatened and system justification. The indicators of individual and collective identity threat were themselves intercorrelated, *r* = .18, *p* < .001, 95% *CI* [.10, .27]. Those with high collective identity threat perception levels were also highly identifying with their roles as teachers. The strong correlation between collective identity threat and resistance to legislative changes, *r* = .59, *p* < .001, 95% *CI* [.53, .64], indicates that these factors tend to occur together, suggesting that higher levels of collective identity threat were associated with higher resistance to legislative changes. Similarly, the non-significant correlation between teacher-role identification and resistance to continuous training, *r* = –.06, *p* = .183, 95% *CI* [–.15, .03], indicates a lack of association between these variables.

### Hypothesis testing

In MPlus we built a model where resistance to legislative changes and continuous training were the output variables, and cognitive conservatism and ideological conservatism were the input variables with the indicator variables of individual identity threat and collective threat the mediators.^2^ Identification with the teacher role was controlled. We used a saturated model that demonstrated a perfect fit, and it was bootstrapped using 1,000 draws to generate 95% confidence intervals. Figures 1a and 1b illustrate the final structural models used to evaluate the hypothesized mediation pathways. Figure 1a presents the model including the cognitive conservatism predictors (Need for closure and Need for cognition), whereas Figure 1b presents the model including the ideological conservatism predictors (Right-wing authoritarianism and Institutional system justification). Only statistically significant standardized paths (*p* < .05) are displayed for clarity; the full model including all paths and covariances is provided in the Supplementary Materials (Figure S1). This approach was employed to determine the significance of the proposed paths within the mediation model. In the SEM, cognitive and ideological conservatism were entered simultaneously as predictors, with their covariance freely estimated to account for shared variance. Finally, to verify that our primary structural paths were not confounded by background characteristics (see Supplementary Table S2) we re-estimated the full SEM including teachers’ political orientation (*r* = –.32, *p* < .001, for resistance to legislative changes; *r* = .14, *p* = .007, for resistance to continuous training), educational or school level (*r* = –.05, *p* = .31, for resistance to legislative changes; *r* = –.14, *p* = .003, for resistance to continuous training), and workplace location (*r* = –.10, *p* = .039, for resistance to legislative changes; *r* = .18, *p* < .001, for resistance to continuous training) as simultaneous covariates predicting both resistance outcomes. None of these predictors reached significance in the SEM (*ps* > .35), and their inclusion altered the original path coefficients by less than *|Δβ|* = .01. Accordingly, we omitted these covariates from the final, parsimonious model in the main text; the results of these analyses are provided in the Supplementary Materials (Tables S3, S4).

**Figure 1a:**
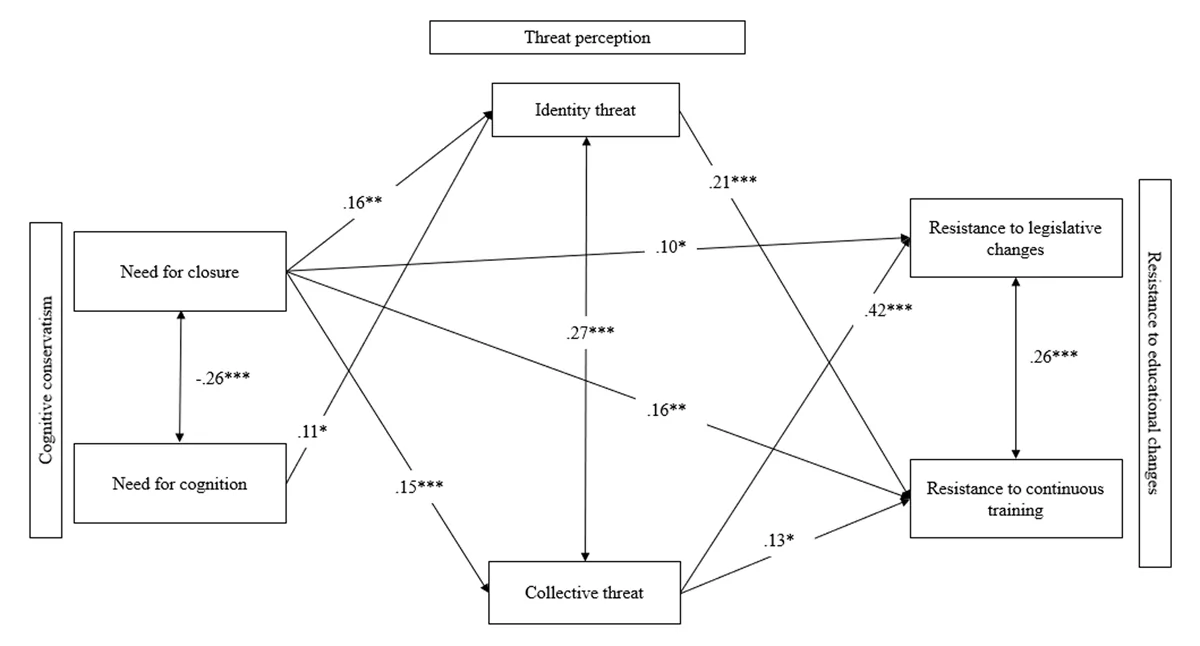
Path model with standardized coefficients for cognitive conservatism predictors of resistance to educational change. *Notes*: All lines indicate direct pathways; ***p < .001, **p < .01, *p < .05.

**Figure 1b:**
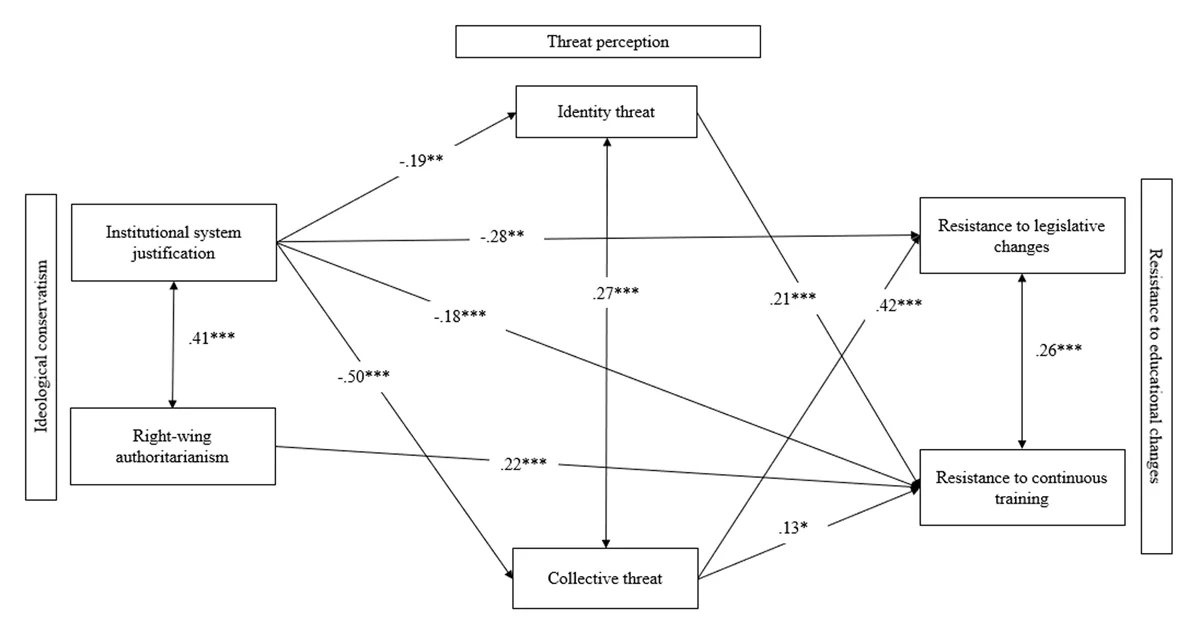
Path model with standardized coefficients for ideological conservatism predictors of resistance to educational change. *Notes*: All lines indicate direct pathways; ***p < .001, **p < .01, *p < .05.

Institutional system justification predicted lower resistance to educational changes for both types of changes examined. Total effects were large and negative for both resistance to legislative changes (*β* = –.50, *SE* = .04, *p* < .001) and resistance to continuous training (*β* = –.28, *SE* = .05, *p* < .001). It also predicted a reduced experience of both collective identity threat (*β* = –.19, *SE* = .06, *p* = .001) and individual identity threat (*β* = –.51, *SE* = .05, *p* < .001). Roughly half of these effects operated indirectly via reduced collective threat (legislative indirect *β* = –.21, 95% *CI*[–.21, –.17], *p* < .001; training indirect *β* = –.07, 95% *CI*[–.11, –.02], p = .015) and, for continuous training only, via reduced individual threat (indirect *β* = –.04, 95% *CI*[–.06, –.02], *p* = .007) (see also Table S2). Right-wing authoritarianism predicted lower resistance to continuous training (direct *β* = .22, *SE* = .05, *p* < .001), but it showed minimal and non-significant indirect effects on resistance to both types of educational changes. In terms of threat, RWA was unrelated to collective identity threat (direct *β* = .09, *SE* = .06, *p* = .107) and to individual identity threat (direct *β* = .004, *SE* = .05, p = .938). Consequently, all of its indirect pathways through both collective and individual threat were non-significant: for legislative resistance via collective threat (indirect *β* = .00, 95% *CI* [–.03, .04], *p* = .939) and via individual threat (indirect *β* = .00, 95% *CI* [–.00, .01], *p* = .511); and for training resistance via collective threat (indirect *β* = .00, 95% *CI* [–.01, .01], *p* = .942) and via individual threat (indirect *β* = .02, 95% *CI* [–.00, .04], *p* = .132). It also did not significantly predict resistance to legislative changes (direct *β* = .01, *SE* = .04, *p* = .795).

Need for closure was associated with greater resistance and heightened threat perceptions, both directly and via threat pathways. It directly predicted higher resistance to legislative changes (direct *β* = .10, *SE* = .04, *p* = .013) and to continuous training (direct *β* = .16, *SE* = .05, *p* = .001), with sizable total effects on both outcomes (legislative total *β* = .17, *p* < .001; training total *β* = .21, *p* < .001).

In the threat domain, need for closure predicted increased collective identity threat (direct *β* = .156, *SE* = .053, *p* = .003) and increased individual identity threat (direct *β* = .149, *SE* = .040, *p* < .001). The impact of need for closure on resistance was also mediated by these heightened threats. Specifically, for legislative resistance, the indirect pathway via collective threat was significant (indirect *β* = .06, 95% *CI* [.03, .09], *p* < .001), whereas the individual-threat pathway was non-significant (indirect *β* = .01, 95% *CI* [–.01, .02], *p* = .479). For training resistance, both mediation paths were significant: via collective threat (indirect *β* = .02, 95% *CI* [.00, .04], *p* = .042) and via individual threat (indirect *β* = .03, 95% *CI* [.01, .05], *p* = .019).

Need for cognition did not predict resistance to legislative changes (total *β* = .01, *p* = .824) or continuous training (total *β* = –.01, *p* = .907), nor did it yield any significant indirect effects via either threat mediator. However, it was positively associated with individual identity threat (*β* = .11, *SE* = .05, *p* = .032) but unrelated to collective identity threat (*β* = .01, *SE* = .05, *p* = .902).

Individual identity threat was a significant predictor of greater resistance to continuous training (*β* = .23, *SE* = .05, *p* < .001), but its association with resistance to legislative changes was non-significant (*β* = .06, *SE* = .03, *p* = .48). Lastly, collective identity threat was a strong, significant predictor of resistance to legislative changes (*β* = .26, *SE* = .05, *p* < .001) and significantly predicted resistance to continuous training (*β* = .23, *SE* = .05, *p* < .001).

To summarize, cognitive and ideological conservatism predicted resistance to educational changes through distinct pathways. Higher institutional system justification predicted lower resistance, both directly and through reduced individual and collective threat perceptions, suggesting that teachers who viewed the system they work in as legitimate were less likely to oppose educational changes. Conversely, higher need for cognitive closure predicted higher resistance to both types of educational change particularly through higher individual and collective threat perception, highlighting the impact of personal discomfort with ambiguity on attitudes toward change. Interestingly, higher right-wing authoritarianism, while directly predicting lower resistance to continuous training, showed no indirect effects through the examined pathways, indicating its broader impact is limited.

## Discussion

This study examined the relationship between cognitive and ideological conservatism, threat perception, and resistance to educational change, using multiple conceptualizations to capture the mechanisms shaping teachers’ responses to constant change. Findings offered mixed support for the hypotheses.

Right-wing authoritarianism (RWA) was positively associated with resistance to continuous training, partially supporting H1. Consistent with prior research (Adorno et al., 1950; Fasce & Avendaño, 2020; Furr et al., 2003; Jost et al., 2003; Jost et al., 2007; Napier & Jost, 2008), high-RWA individuals may resist even ideologically aligned conservative reforms when these disrupt trusted structures. In contrast, institutional system justification was negatively associated with both resistance types, aligning with its palliative function (Hadarics et al., 2021; Jost et al., 2007; Napier et al., 2020): viewing the system as just reduces perceptions of professional identity threat and fosters acceptance of top-down legislative changes. This highlights a key difference within ideological conservatism: system justifiers accept legitimate-seeming changes, while high-RWA individuals resist change due to a need for stability, regardless of ideological direction.

Aligning with H1, need for closure predicted higher resistance to both change types, likely because legislative and training demands threaten epistemic clarity (Kruglanski et al., 1993). Need for cognition was not directly related to resistance, though it predicted higher individual professional identity threat.

The second hypothesis (H2) was partially supported: both individual and collective identity threat predicted greater resistance, consistent with motivated social cognition theory, which links fear and uncertainty to resistance (Duckitt, 2001; Jost et al., 2003; Jost et al., 2007).

The third hypothesis (H3) also received partial support: identity threat mediated some, but not all, relationships between conservatism and resistance. System justification reduced resistance to legislative and training requirements by lowering threat perceptions, even in a sample with low overall system justification. Conversely, need for closure increased resistance primarily through heightened threat perceptions. Identity threat may strongly influence resistance only when change is perceived as destabilizing – more likely for cognitive conservatism, which values stability and routine, and less so for system justification, which may dampen threat perceptions even when changes are disadvantageous. Overall, ideological conservatism (e.g., system justification) was generally linked to lower resistance, while cognitive conservatism (e.g., need for closure) predicted greater resistance. This divergence may explain the partial mediation: for system justifiers, aligned reforms may not trigger threat, whereas for those high in need for closure, even ideologically aligned reforms can provoke threat if they disrupt routines or increase uncertainty. Thus, practical consequences of reform can trigger identity-based resistance regardless of ideological alignment.

## Limitations

This study has several limitations. A methodological limitation concerns the relatively low internal consistency of several measures. Specifically, the two subscales of the Resistance to educational change questionnaire (Lomba-Portela et al., 2022) – Resistance to legislative changes (*α* = .68) and Resistance to continuous training (*α* = .67) – showed slightly below-optimal reliability. Among the ideological predictors, the 3-item Right-Wing Authoritarianism Scale (Heller et al., 2020) had an *α* of .63, and the 4-item Identification with the teacher role scale (adapted from Doosje et al., 1995) also yielded *α* = .63. While these coefficients are acceptable for short multi-item measures frequently used in large-scale survey research, they nevertheless indicate a degree of measurement imprecision. To assess structural validity, we conducted a confirmatory factor analysis on the two resistance subscales, which supported the expected two-factor structure with adequate model fit (*χ²* = 94.94, *df* = 33, *p* < .001; TLI = .92; CFI = .89; RMSEA = .06; SRMR = .05). These moderate reliability values may have attenuated the observed path coefficients, leading to conservative estimates of associations rather than inflated effects. However, the measures demonstrated theoretically consistent intercorrelations and factor structures, providing support for their conceptual validity. Future studies should consider using longer or psychometrically refined versions of these scales to enhance reliability and replicability.

Another limitation concerns the Institutional System Justification Scale, which was developed specifically for this study to capture the perceived legitimacy of the Hungarian educational system. Although the scale demonstrated good internal consistency (*α* = .84) and theoretical coherence, its construct validity has not yet been established beyond the present context. Therefore, interpretations of the findings involving this measure should be treated with caution, and future research should aim to replicate and validate this scale in independent samples and cultural settings. Additionally, the use of a convenience sample reduces external validity: vocational and technical school teachers were underrepresented, and those with strong opinions may have been more likely to participate. Future research should apply stratified or random sampling across school types, regions, age groups, and political orientations for better representativeness.

The cross-sectional design precludes causal inference, leaving the temporal order of variables uncertain. While the findings align with theoretical expectations, other unmeasured factors may influence the observed associations.

Finally, participants may have interpreted resistance mainly through practical rather than ideological considerations. Although the reforms were ideologically conservative, they may have been experienced chiefly as professional disruptions (e.g., increased workload, reduced autonomy). Ideological orientations such as system justification or right-wing authoritarianism may thus affect resistance only when the ideological character of reforms is consciously recognized, potentially explaining differences across ideological constructs.

## Conclusion

This study demonstrates that conservatism is not a monolithic construct in predicting responses to social and institutional change. Regarding the paradoxical situation of the reaction of conservative individuals to conservative educational changes, we found that teachers high in right-wing authoritarianism and those with a strong need for closure both tend to resist changes, even conservative ones, because they perceive such changes as disruptions to the established practices and certainty they value. In contrast, lower resistance was associated with institutional system justification, which reduced perceived threats and increased acceptance of the status quo. Rather than uniformly promoting resistance, distinct conservative motives operate in opposing directions: cognitive conservatism – rooted in a need for certainty and structure – intensifies resistance, whereas ideological conservatism, expressed through system-justifying beliefs, can reduce resistance by legitimizing authority and stability. Recognizing this duality helps to explain why individuals who are otherwise motivated to preserve order may still resist conservative reforms that threaten their professional or epistemic security.

Our study adds to the existing body of literature by highlighting the nuanced ways in which different facets of conservatism – cognitive and ideological – interact with identity threat perception to influence resistance to educational change. Previously, it was understood that conservatism in general contributes to resistance to change; but this research adds a new element to our understanding of resistance to change, by employing individual and collective identity threat awareness as mediator variables. We presented evidence of their relationships with cognitive and ideological conservatism. This study therefore substantiates the theory that the need to manage uncertainty and threat is closely linked with conservatism (as suggested by Jost et al., 2007) and it also provides insights into why teachers in precarious positions are inclined to accept changes that may disadvantage them (Napier, Bettinsoli, & Suppes, 2020).

The palliative effect of system justification is underpinned by the fact that these changes have placed the teaching population in a more precarious position than prior to the changes. Therefore, it is a strategy to reduce fear and threat by adopting an attitude where changes are more readily accepted when the authority behind them is deemed legitimate, even if the changes harm the population. This helped us deepen our understanding about the psychological mechanisms that influence teachers’ reactions to institutional changes and clarify the complex motivations behind resistance behaviours in educational settings. Future research should further explore the psychological and contextual conditions under which identity threat functions as a key mediator in resistance to educational change. While this study focused on threat-related mechanisms, it remains plausible that other factors – such as institutional trust, perceived procedural justice, value conflict, burnout, perceived legitimacy of reform efforts, levels of professional autonomy, or emotional exhaustion – may also shape how teachers respond to institutional changes. Investigating these additional pathways could provide a more comprehensive understanding of the dynamics underlying resistance and clarify when conservatism leads to resistance through threat sensitivity and when other motivational or structural forces dominate. Longitudinal or experimental designs may also help disentangle causal directions between perceived threat, belief systems, and resistance behaviour.

Finally, although each social context is unique, the conservative wave shaping Hungary’s educational system has parallels worldwide. In Poland, the 2017 PiS reform dismantled the three-tier system without consulting teachers, sparking nationwide strikes, while the later ‘Lex Czarnek’ package placed schools under politically appointed regional heads, reduced teacher autonomy, and standardized curricula and textbooks (Ustawa o zmianie ustawy – Prawo oświatowe, 2017, Ustawa z dnia 8 grudnia 2021 r. o zmianie ustawy – Prawo oświatowe oraz niektórych innych ustaw, 2021). In the United States the Trump administration issued executive orders limiting diversity training and prohibiting certain content in K–12 schools, criticized by educators’ organizations and free speech advocates (Combating Race and Sex Stereotyping, 2020, Ending Radical Indoctrination in K-12 Schooling, 2025).

These examples – from rapid centralization to restrictive content policies – mirror the psychological dynamics documented here, underscoring the importance of understanding teachers’ responses. As a large and influential professional group, teachers play a crucial role in shaping the perspectives of future generations.

## Additional File

The additional file for this article can be found as follows:

10.5334/irsp.1041.s1 Supplementary Information. Supplementary Information contains the full item wording of all adapted and newly developed scales (S1), total, direct, and indirect effects for the structural model (Table S2), bivariate correlations including background covariates (Table S3), regression paths for covariates (Table S4), and the complete SEM path model with all estimated parameters (Figure S1).

## Data Availability

The databases of this research have been uploaded to the OSF site and they are available for blind review: https://osf.io/6ctsx/?view_only=15770b26ef5c4866a74d3fc07f9da00f.

## Footnotes

Act CLXXXVIII of 2012 on the State Takeover of certain institutions providing public education and maintained by municipalities (local governments).

Although our preregistration specified an interaction between conservatism and identity threat, the results did not support this approach, as the interaction term was non-significant. In light of these findings, we adopted an alternative, theory-driven model in which identity threat operates as a mediator between conservatism and resistance to change. This shift is consistent with prior research conceptualizing threat perception as a cognitive-affective mechanism linking conservative beliefs to resistance (e.g., Jost et al., 2007). In line with this conceptual shift, the preregistered hypotheses were also reformulated to reflect competing predictions among the conservatism-related variables (see Aims and Objectives section).
