# Supplementary material for: The Conservatism Paradox: Conservatism Among Teachers Predicts Resistance Even to Conservative Change
Source: Int Rev Soc Psychol. 2026 Nov 3;39:12. doi: 10.5334/irsp.1041 (PMC13741931; doi:10.5334/irsp.1041)
Supplement: Supplementary file 1 [file irsp-39-1041-s1.pdf]

The Conservatism Paradox: Conservatism among Teachers predicts Resistance even to Conservative change

Supplementary Information

Table of Content

S1. Measures 2

Table S2. Total, indirect and direct effects of the indicator variables of cognitive and ideological conservatism on the two types of resistance to educational change 7

Table S3. Correlations Between Study Variables and Covariates 1

Table S4. Path Coefficients from Covariates to Resistance Outcomes 2

Figure S1. Path model with standardized coefficients for the relationships between the indicator variables of cognitive and ideological conservatism, threat perception and resistance to educational changes 2

S1. Measures

1. *Resistance to educational change*

How much do you agree with the following statements?

- Legislative changes have negative effects on teaching attitudes.
- With the successive educational changes, I am afraid of losing confidence in my daily work performance.
- With the educational changes I think “the cure will be worse than the disease”.
- It is disconcerting to have to change since it is decided by others.
- The tentativeness of educational change undermines my commitment as a teacher.
- The teacher plays too many roles.
- The training courses do not provide me with relevant content.
- Continuous training is pure theory.
- Theory is one thing, but practice is quite another.
- The integration of students with special educational needs causes a decrease in the level of performance in the classroom.

2. *Need for closure*

Please read each of the statements below carefully and decide how much you agree with each statement based on your opinions and experiences.

- I don't like situations that are uncertain.
- I dislike questions which could be answered in many different ways.
- I find that a well ordered life with regular hours suits my temperament.
- I feel uncomfortable when I don't understand the reason why an event occurred in my life.
- I feel irritated when one person disagrees with what everyone else in a group believes.
- I don't like to go into a situation without knowing what I can expect from it.
- When I have made a decision, I feel relieved.
- When I am confronted with a problem, I'm dying to reach a solution very quickly.
- I would quickly become impatient and irritated if I would not find a solution to a problem immediately.
- I don't like to be with people who are capable of unexpected actions.
- I dislike it when a person's statement could mean many different things.
- I find that establishing a consistent routine enables me to enjoy life more
- I enjoy having a clear and structured mode of life.
- I do not usually consult many different opinions before forming my own view.
- I dislike unpredictable situations.

3. *Need for cognition*

How well do the following ways of thinking describe you?

- I try to anticipate and avoid situations where there is likely a chance I will have to think in depth about something.

- I really enjoy a task that involves coming up with new solutions to problems.
- The notion of thinking abstractly is appealing to me.
- It's enough for me that something gets the job done; I don't care how or why it works.
- I usually end up deliberating about issues even when they do not affect me personally.

4. *Right-wing authoritarianism*

Thinking about society, how much do you agree with the statements below?

- Troublemakers should clearly feel the effects of the fact that they are unwanted in the society.
- People should leave important decisions to those in charge/the leaders.
- Established conducts should not be questioned.

5. *Institutional system justification*

Thinking about the Hungarian education system, to what extent do you agree with the following statements?

- The Hungarian education system must be transformed.
- The Hungarian education system is the best in the world.
- The Hungarian education system is generally good.
- Both children and teachers have a chance to succeed in the Hungarian education system.
- The Hungarian education system works well.
- The Hungarian education system is getting worse every year.
- Everyone involved in the Hungarian education system usually gets what they deserve.

6. *Individual professional identity threat*

On a scale of 1 („Disagree”) to 5 („Agree”), please indicate how much you agree or disagree with each statement. There are no right or wrong, correct or incorrect answers.

- It is quite easy for a teacher to lose the respect that a teacher deserves.
- There are teachers who never become teachers (with a capital letter), no matter how long they have been teaching.
- People often question whether someone is a real, genuine teacher based on the quality of their work or their methods.
- People often question whether someone is a real, genuine teacher, based on their personality.
- Being a teacher is not a given - it is easy for a teacher to end up not being seen as a real teacher.

7. *Collective professional identity threat*

Please indicate your level of agreement with each statement on a scale of 1 to 5.

- I am afraid because teachers are very vulnerable at the moment.
- I am afraid that teachers will be attacked.
- I fear that too few people support teachers' goals.
- I fear that teachers will not achieve their goals.
- I am concerned that the future of the teaching profession is at risk.

8. *Identification with the teacher role*

Thinking about your identity as a teacher, to what extent do you agree with the following statements?

- I identify with the other teachers.
- I see myself as a teacher.

- I am happy to be a teacher.
- I have strong ties to the teaching community.

9. Other possible covarying variables that were measured with question/scales

1. *Political orientation:* Numeric; “People often locate their political orientation on a left–right spectrum. Please place yourself on this scale.” Self-placement on a left–right political orientation scale (1 = Left, 10 = Right)
2. *Profession:* Numeric; What is your current teaching role?
    a. 1 = Kindergarten teacher
    b. 2 = Lower-grade primary (grades 1–4)
    c. 3 = Upper-grade primary (grades 5–8)
    d. 4 = Secondary/high school teacher
    e. 5 = Higher education teacher
    f. 6 = Special-needs teacher
    g. 7 = Other
3. *Status:* Numeric; What is your current pedagogical rank?
    a. 1 = Intern
    b. 2 = Teacher I
    c. 3 = Teacher II
    d. 4 = Senior teacher
    e. 5 = Master teacher
    f. 6 = Other
4. *Institution type:* Numeric; Are you currently teaching, and if so, in which institution type?
    a. 1 = Primary grades 1–4

   b. 2 = Primary grades 5–8
   c. 3 = Vocational school
   d. 4 = General high school
   e. 5 = Other
   f. 6 = Not currently teaching
5. *Location:* Numeric; Location of your school/workplace (1 = Capital, 2 = County seat, 3 = City, 4 = Municipality)

Table S2. Total, indirect and direct effects of the indicator variables of cognitive and ideological conservatism on the two types of resistance to educational change

	B	S.E.	p	95% CI	
				Lower	Upper
Effects from Institutional system justification to Resistance to legislative changes					
Total	-.50***	.04	**<.001**	-.57	-.43
Total indirect	-.22***	.03	**<.001**	-.22	-.17
Specific indirect					
Institutional system justification → Identity threat → Resistance to legislative changes	-.01	.01	.461	-.02	.01
Institutional system justification → Collective threat → Resistance to legislative changes	-.21***	.03	**<.001**	-.21	-.17
Direct	-.28***	.05	**<.001**	-.28	-.21
Effects from Right-wing authoritarianism to Resistance to legislative changes					
Total	.02	.05	.742	-.06	.10
Total indirect	.01	.02	.839	-.03	.04
Specific indirect					
Right-wing authoritarianism → Identity threat → Resistance to legislative changes	.00	.00	.511	-.00	.01
Right-wing authoritarianism → Collective threat → Resistance to legislative changes	.00	.02	.939	-.03	.04
Direct	.01	.04	.795	-.00	.09
Effects from Need for closure to Resistance to legislative changes					
Total	.17***	.04	**<.001**	.10	.24
Total indirect	.07***	.02	**<.001**	.04	.10
Specific indirect					

Need for closure → Identity threat → Resistance to legislative changes	.01	.01	.479	-.01	.02
Need for closure → Collective threat → Resistance to legislative changes	.06***	.02	**<.001**	.03	.09
Direct	.10*	.04	**.013**	.03	.17
Effects from Need for cognition to Resistance to legislative changes					
Total	.01	.04	.824	-.06	.08
Total indirect	.01	.02	.776	-.03	.04
Specific indirect					
Need for cognition → Identity threat → Resistance to legislative changes	.00	.01	.485	-.01	.01
Need for cognition → Collective threat → Resistance to legislative changes	.00	.02	.903	-.03	.04
Direct	.00	.04	.920	-.06	.07
Effects from Institutional system justification to Resistance to continuous training					
Total	-.28***	.05	**<.001**	-.36	-.20
Total indirect	-.10***	.03	**<.001**	-.15	-.06
Specific indirect					
Institutional system justification → Identity threat → Resistance to continuous training	-.04**	.01	**.007**	-.06	-.02
Institutional system justification → Collective threat → Resistance to continuous training	-.07*	.03	**.015**	-.11	-.02
Direct	-.18**	.05	**.001**	-.27	-.10
Effects from Right-wing authoritarianism to Resistance to continuous training					
Total	.24***	.05	**<.001**	.15	.32
Total indirect	.02	.02	.221	-.01	.05
Specific indirect					
Right-wing authoritarianism → Identity threat → Resistance to continuous training	.02	.01	.132	-.00	.04
Right-wing authoritarianism → Collective threat → Resistance to continuous training	.00	.01	.942	-.01	.01
Direct	.22***	.05	**<.001**	.13	.30
Effects from Need for closure to Resistance to continuous training					
Total	.21***	.05	**<.001**	.13	.30
Total indirect	.05**	.02	**.002**	.02	.08
Specific indirect					
Need for closure → Identity threat → Resistance to continuous training	.03**	.01	**.019**	.01	.05
Need for closure → Collective threat → Resistance to continuous training	.02*	.01	**.042**	.00	.04
Direct	.16***	.05	**.001**	.08	.23

Effects from Need for cognition to Resistance to continuous training					
Total	-.01	.05	.907	-.08	.07
Total indirect	.02	.02	.137	-.00	.05
Specific indirect					
Need for cognition → Identity threat → Resistance to continuous training	.02	.01	.068	.00	.05
Need for cognition → Collective threat → Resistance to continuous training	.00	.01	.906	-.10	.01
Direct	-.03	.04	.498	-.17	.04

Table S3. Correlations Between Study Variables and Covariates

Variables	1.	2.	3.	4.	5.	6.	7.	8.	9.	10.	11.	12.	13.	14.	15.
1. Legislative changes	–														
2. Continuous training	.33**	–													
3. Need for closure	.12*	.26**	–												
4. Need for cognition	.06	–.09*	–.26**	–											
5. Right-wing authoritarianism	–.13**	.20**	.39**	–.29**	–										
6. Institutional system justification	–.48**	–.15**	.12**	–.18**	.41**	–									
7. Individual identity threat	.23**	.30**	.14**	.08	–.15**	–.49**	–								
8. Collective identity threat	.59**	.25**	.10*	.07	.04	–.07	.33**	–							
9. Identification with the teacher role	.14**	–.06	.08	.13	.10*	–.05	.05	.18**	–						
10. Age	–.06	.00	.03	.05	.04	.06	.07	.16	.17	–					
11. Political orientation	–.32**	.14**	–.21**	–.15*	.38**	.41**	–.33**	.06	–.10	–.09	–				
12. Profession	–.02	–.24**	–.06	.05	–.09	.01	–.07	–.10	–.03	.08	–.12*	–			
13. School level	–.05	–.14**	–.17**	.17**	–.04	.35**	–.04	–.03	.16**	.20*	–.04	.09	–		
14. Institution type	–.01	–.18**	–.15**	.17**	–.12*	.25**	.03	–.11*	.01	–.07	–.13**	.65**	.06	–	
15. Location	–.10*	.18**	.11*	–.06	.27**	.90**	–.04	.02	.03	.15*	.21**	–.26**	.91**	–.32**	–
16. Experience	–.03	–.03	.07	.06	.09	.03	–.06	–.05	.20**	.15	–.09	–.09	.35**	–.05	.10*

Notes **p* < .05; ***p* < .001

Table S4. Path Coefficients from Covariates to Resistance Outcomes

Predictor	Outcome	ß	SE	p
Political orientation	Resistance to legislative changes	0.02	0.04	.65
Political orientation	Resistance to continuous training	0.01	0.03	.75
Institution type	Resistance to legislative changes	–0.01	0.05	.80
Institution type	Resistance to continuous training	0.00	0.04	.92
Location	Resistance to legislative changes	0.00	0.03	.87
Location	Resistance to continuous training	–0.01	0.02	.70

Note. All effects are standardized path coefficients (β), with SE = standard error; none of the covariates reached significance at p < .05.

Figure S1. Path model with standardized coefficients for the relationships between the indicator variables of cognitive and ideological conservatism, threat perception and resistance to educational changes

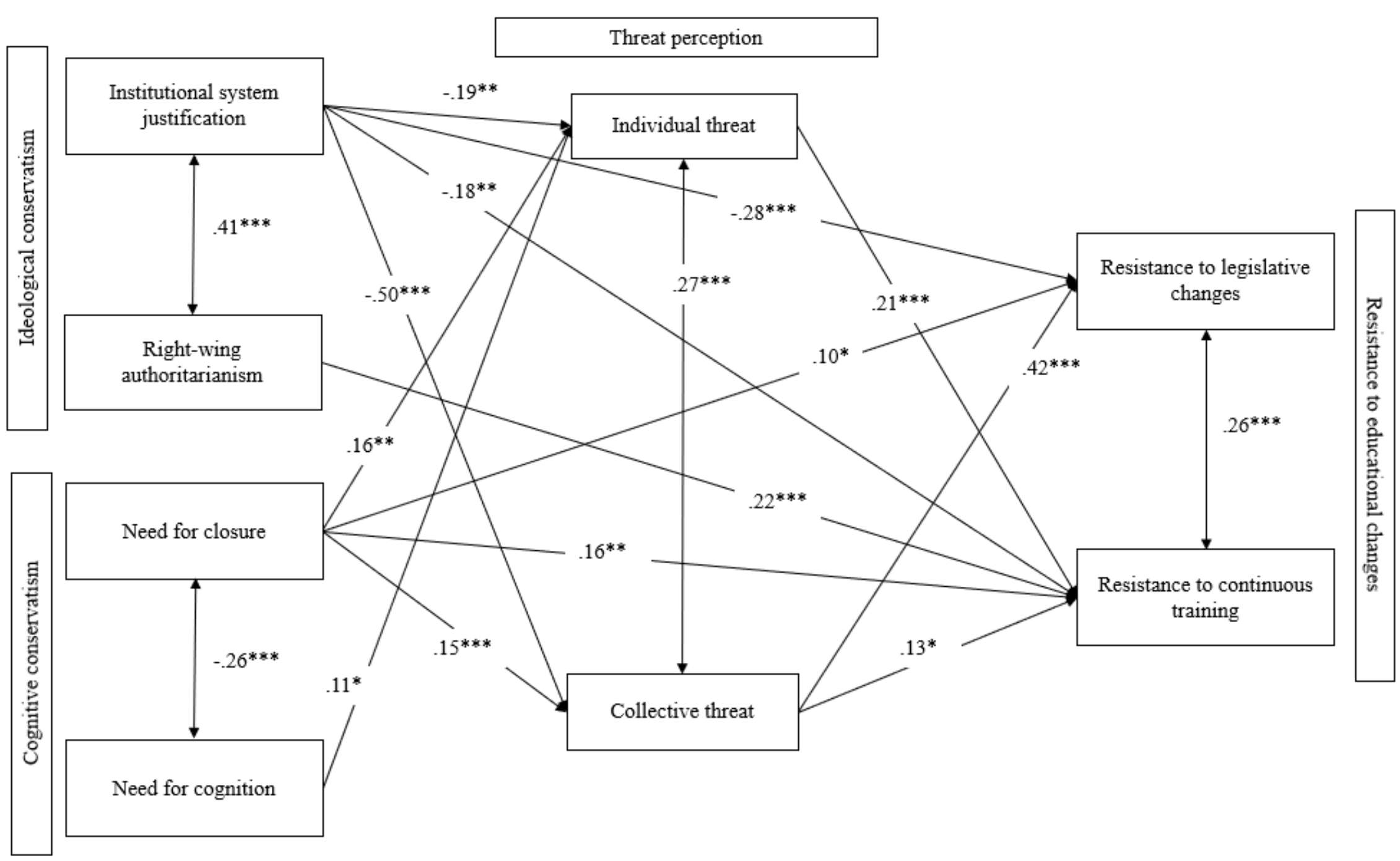

Notes All lines indicate direct pathways; ***p < .001, **p < .01, *p < .05
